# Food neophobia and mealtime food consumption in 4–5 year old children

**DOI:** 10.1186/1479-5868-3-14

**Published:** 2006-07-06

**Authors:** Lucy Cooke, Susan Carnell, Jane Wardle

**Affiliations:** 1 Cancer Research UK Health Behaviour Unit, Department of Epidemiology and Public Health, University College London, UK

## Abstract

**Background:**

Previous research has documented a negative association between maternal report of child food neophobia and reported frequency of consumption of fruit, vegetables, and meat. This study aimed to establish whether neophobia is associated with lower intake of these food types in naturalistic mealtime situations.

**Methods:**

One hundred and nine parents of 4–5 year olds completed questionnaires which included a six-item version of the Child Food Neophobia Scale (CFNS). The children took part in a series of 3 test lunch meals at weekly intervals at school at which they were presented with: chicken, cheese, bread, cheese crackers, chocolate biscuits, grapes and tomatoes or carrot sticks. Food items served to each child were weighed before and after the meal to assess total intake of items in four categories: Fruit and vegetables, Protein foods, Starchy foods and Snack foods. Pearson Product Moment Correlations and independent t tests were performed to examine associations between scores on the CFNS and consumption during lunches.

**Results:**

Neophobia was associated with lower consumption of fruit and vegetables, protein foods and total calories, but there was no association with intake of starch or snack foods.

**Conclusion:**

These results support previous research that has suggested that neophobia impacts differentially on consumption of different food types. Specifically it appears that children who score highly on the CFNS eat less fruit, vegetables and protein foods than their less neophobic peers. Attempts to increase intake of fruit, vegetables and protein might usefully incorporate strategies known to reduce the neophobic response.

## Background

Food neophobia is defined as avoidance of, and reluctance to taste, unfamiliar foods [1,2]. It might be assumed that such a behaviour pattern would have negative dietary consequences in terms of the variety of foods consumed although logically, a highly neophobic child need not have a limited dietary repertoire provided that he/she has previously been familiarised with a wide range of foods. In recent years there has been increasing interest in documenting the impact of neophobia on the quality and variety of young children's diets.

Some researchers have investigated the relationship between food neophobia and the *number *of foods served by parents or tried, liked and disliked by children. In two studies of Swedish families with children aged from 2 to 17 years old, Koivisto Hursti and colleagues found that higher neophobia was associated with fewer uncommon foods being served and fewer foods being tried [3,4]. Likewise, a study of North American 2–8 year olds found that neophobia was negatively related to the number of foods liked and positively related to the number of foods disliked [5].

Other studies have examined the relationship between neophobia and consumption of specific food types. In the USA a study of seventy 9–10 year olds examined energy and nutrient intake as well as consumption of servings from the major food groups and found that neophobic children consumed more saturated fat and had less dietary variety [6] although they did not differ from average or neophilic children in number of servings of any food group consumed. However, the small sample size in this study may have limited its power to detect differences.

In a large-scale survey of the eating behaviours of 2–6 year old British children, we found that parent-reported frequency of fruit and vegetable consumption was strongly inversely related to child food neophobia [7]. This inverse relationship also held for meat and fish consumption, but not for starchy foods or for sweet or fatty snack foods [8].

The only other study to examine the relationship between child food neophobia and intake of specific food items is noteworthy because of its inclusion of an additional measure of pickiness [9]. Parents of 7-year-old girls completed measures of both characteristics as well as a measure of vegetable consumption. When considered separately, neophobia and pickiness were inversely related to vegetable consumption and girls who were both 'picky' *and *neophobic ate significantly fewer vegetables than those who were neither. Definitions of pickiness vary somewhat, but because behaviours associated with the label include rejection of certain foods or food types and acceptance of a very limited range of foods, considerable overlap with the construct of neophobia seems plausible. However, neophobia and pickiness were only modestly correlated in this sample and the authors concluded that the two are distinct behavioural concepts.

Although limited, the research that has been undertaken in this area points to a detrimental effect of neophobia on children's eating habits. Specifically, it appears that children who are more neophobic may eat less fruit and vegetables, more fat and less varied diets than their more neophilic peers. However, a limitation of research to date is that both child food neophobia and food consumption have been assessed using parent report which may be susceptible to social desirability bias. In addition, respondents may be affected by a drive for consistency in their answers, for example, having reported that their child ate certain "healthy" foods infrequently, they might respond more negatively to items concerning their child's willingness to try foods. It is important to establish whether food neophobia impacts on consumption of these food types measured objectively in real mealtime situations.

The aim of the present study was to investigate the relationship between CFNS scores and food intake in 4–5 year olds during specially prepared school lunches. Based on previous findings, we predicted that higher scores would be negatively related to consumption of fruit, vegetables and meat/foods of animal origin but unrelated to intake of starchy or snack foods.

## Methods

### Participants and procedure

Children aged 4 to 5 years old in five classes at four London primary schools were recruited to a study of children's eating behaviour. Information sheets, consent forms and questionnaires were sent home to parents. Those who wished to participate completed consent forms and questionnaires and returned both to their child's class teacher. The questionnaire included the six-item version of the Child Food Neophobia Scale (CFNS; [10]) that we have used in previous research [7,8]. The original CFNS is a 10-item scale to measure children's willingness to sample new foods, scores on which correlate highly with behavioural measures of neophobia. Four items were excluded on the basis that they were innapropriate for the age range of our sample (e.g. "My child likes to eat in ethnic restaurants). The six remaining items were: "My child does not trust new foods", "If my child doesn't know what's in a food, s/he won't try it", "My child is afraid to eat things s/he has never had before", "My child will eat almost anything" (reverse scored), "My child is very particular about the foods s/he will eat" and "My child is constantly sampling new and different foods" (reverse scored). Responses are on a 4 point scale from 'strongly disagree' to 'strongly agree'. Higher scores indicate higher neophobia. Cronbach's alpha for the 6-item version was 0.92 in this sample.

Children's food intake was recorded over 3 'test' lunchtime meals at school at weekly intervals. The study was part of a three-day repeated measures study of intake regulation, so on two out of the three days (Days 2 and 3), children were given a 200 ml orange squash drink prior to lunch. On one day this squash contained 5 kcal and on the other day the squash contained 174 kcal; drink conditions were counter-balanced across days. On Day 1 children received no preload drink, forming a control condition. Children participated in all conditions providing they were present on the day in question. Actions taken to account for the preload design are described in the Results section.

The test meal comprised weighed portions of chicken slices, cheese, bread roll with margarine, cheese crackers, chocolate biscuits, grapes and tomatoes or carrot sticks, served cold and presented to each child in an individual compartmented container. Tomatoes had been planned for all meals, but in the first class visited, a large number of children reported disliking tomatoes, so children in the remaining 4 classes were given sticks of raw carrot instead. To maintain consistency, children in the first class were given tomatoes at all subsequent meals. Children were not required to 'clean their plates, but were told to eat as much or as little of the meal as they wished.

Extra pre-weighed portions of bread rolls were offered when children had finished their original servings and unlimited water was provided. Children were observed throughout the meals in order to ensure that food was not shared. Children raised their hand to indicate when they had finished their meal and containers were then collected by researchers and sealed. Foods were subsequently weighed to establish intake in grammes of each food type. Energy content (kcal) of processed foods (chicken slices, cheese slices, cheese crackers, and chocolate biscuits) was calculated using manufacturers' information. McCance and Widdowson's 'The Composition of Foods' [11] was used to calculate energy content of the remaining foods (bread, grapes, tomatoes and carrots).

## Results

### Response rates

Of the 149 children eligible to take part in the study, 120 were present on at least one of the three days of the study. Questionnaires were returned by 90.8% (109/120) of the participating children's parents and data from this group are used here to analyse the relationship between neophobia and food intake.

### Participant characteristics

Among the participating children for whom questionnaire data were available (n = 109), 50.5% were male and 49.5% female, and the mean age was 5.0 years (SD 0.39). Characteristics of parents who completed questionnaires (n = 109) are presented in Table 1. Almost all were mothers and most were reasonably affluent, well-educated, white homeowners.

**Table 1:** Parent characteristics (n = 109)

	**n**	**%**
**Gender**		
Male	3	2.8
Female	106	97.2

**Education**		
None	1	0.9
GCSE or equivalent	31	28.4
A level or equivalent	27	24.7
Degree level or higher	45	41.4
Other	3	2.8
Missing	2	1.8

**Ethnicity**		
White Caucasian	82	75.2
South Asian (Indian/Pakistani)	13	11.9
Black (African/Caribbean)	11	10.1
Other	3	2.8

**Home ownership**		
Own home/buying	95	87.2
Renting	10	9.2
Other	3	2.7
Missing	1	0.9

**Annual household income**		
< £30,000.	24	22.0
£30,000 – £59,999	36	33.0
> £60,000	17	15.6
Missing	32	29.4

### Child food neophobia

Children's mean score on the CFNS was 1.64 (s.d. 0.73). Boys scored higher than girls (boys: 1.75, s.d. 0.80, girls: 1.52, s.d. 0.63) but this difference was not statistically significant. Neophobia did not vary according to child age, or SES as indexed by parental education level, home ownership or annual household income. There were also no differences in neophobia between white children (n = 84) and those from other ethnic groups (n = 25).

### Food intake

Average meal intake of individual items was calculated. Where data were missing for one or more days, means were based on the remaining days. In the majority of cases (73.4%), no data were missing; 23 cases (21.1%) had one day missing and 6 cases (5.5%) had two days of data missing. The magnitude of the associations in the main correlational analyses were unchanged when incomplete cases were excluded so analyses with the full sample were used to increase power and are reported here.

Table 2 gives means and standard deviations (in grammes and kilocalories) for intake of individual food items and for total calorie intake. Means for tomatoes and carrots are based on reduced numbers because tomatoes were offered only in one school class. Means for chicken and cheese exclude data from four vegetarians who were given two portions of cheese, but no chicken.

**Table 2:** Descriptive statistics for food items offered and consumed over three test days (n = 109)

**Food item**	**Mean grams offered (s.d.)** ***range***	**Mean kcal offered (s.d.)** ***range***	**Mean grams consumed (s.d.)** ***range***	**Mean kcal consumed (s.d.)** ***range***
Tomatoes^1^	99.8 (4.62) *87.1 – 105.1*	18.0 (0.83) *15.7 – 18.9*	5.5 (13.84) *0 – 51.8*	1.0 (2.49) *0 – 9.3*
Carrots^2^	54.2 (0.91) *52.0 – 56.6*	19.0 (0.32) *18.2 – 19.8*	9.3 (12.98) *0 – 54.51*	3.3 (4.54) *0 – 19.1*
Grapes	98.7 (3.19) *93.0 – 110.1*	63.7 (2.06) *60.0 – 71.0*	30.8 (32.05) *0 – 105.2*	19.9 (20.66) *0 – 67.8*
Bread rolls	53.6 (5.13) *44.1 – 73.5*	143.6 (13.74) *118.1 – 197.0*	27.4 (18.40) *0.3 – 73.5*	73.4 (49.30) *0.9 – 197.0*
Mini cheese biscuits	36.2 (2.78) *33.5 – 48.6*	191.6 (14.72) *177.4 – 257.0*	14.5 (11.31) *0 – 36.3*	76.6 (59.81) *0 – 192.2*
Mini chocolate biscuits	50.5 (2.08) *35.6 – 53.1*	260.4 (10.75) *183.5 (274.0)*	33.7 (15.00) *0 – 52.4*	179.0 (82.49) *0 – 270.4*
Chicken^3^	63.6 (3.43) *42.0 – 72.7*	74.4(4.01)* 49.1 – 85.1*	23.9 (19.88) *0.5 – 76.5*	27.9 (23.26) *0.5 – 76.5*
Cheese^4^	48.4 (2.54) *43.3 – 64.1*	198.5 (10.42) *177.4 – 262.9*	16.3 (15.30) *0 – 52.6*	66.8 (62.73) *0 – 215.7*
Total	410.5 (21.89) *368.1 – 475.4*	954.5 (33.79) *852.8 – 1085.4*	155.3 (63.88) *55.4 – 396.0*	442.8 (158.37) *62.5 – 925.8*

^1^n = 14, ^2^n = 95

^3^n = 105 (excluding 4 vegetarians, who were not offered chicken)

^4^n = 105 (excluding 4 vegetarians, who were offered an extra portion of cheese to replace their portion of chicken)

Individual food items were combined into four groups reflecting their role in a balanced diet: grapes and carrots/tomatoes (Fruits and vegetables), chicken and cheese (Protein foods), bread rolls (Starchy foods) and chocolate biscuits and cheese crackers (Snack foods). Consumption of foods consumed *within *categories was calculated. Distributions of intake of several individual items and food groups were slightly skewed, but repeating the analyses either using non-parametric tests or with log transformed variables made no difference to the findings. The results of parametric analyses are therefore reported here for consistency with the descriptive data.

Independent t tests were used to examine gender differences. Boys' intake of chocolate biscuits was significantly greater than that of girls' (t(107) = 3.46, p < 0.01), but no other gender differences were observed. Further analyses were therefore carried out on the whole sample.

### Relationship between neophobia and food consumption

Pearson product moment correlations were calculated to examine the relationship between scores on the CFNS and consumption (in grams) of each of the four food categories and total kilocalories consumed (see Table 3). Adjusting for child age, SES variables and ethnicity made no difference to results.

**Table 3:** Correlations between scores on the Child Food Neophobia Scale (CFNS) and consumption of food during school lunches, grouped by type

**Intake**	**Correlation with CFNS score**
**Fruits & vegetables:**	
Grapes and tomatoes/carrots (g)	-0.27**
**Protein foods:**	
Chicken and cheese (g)	-0.34**
**Snack foods:**	
Chocolate & cheese biscuits (g)	0.04
**Starch:**	
Bread rolls (g)	-0.13
**Total calories**	-0.23*

**p < 0.01, *p < 0.05

Neophobia was associated with significantly lower consumption of grapes and tomatoes/carrots and of chicken and cheese. In addition, children who were more neophobic consumed fewer calories overall. Neophobia was not significantly related to intake of bread rolls or snacks. In order to check whether differences in intake patterns simply reflected the fact that neophobic children consumed less calories overall, we re-calculated correlations between neophobia and each food group controlling for overall intake. The inverse correlations between neophobia and both 'fruit and vegetables' and protein foods were slightly reduced but remained significant. The small positive correlation between snack intake and neophobia increased slightly, but still did not reach statistical significance. We therefore report only the unadjusted analyses.

We also wished to test for any effects of the preload, and so the correlational analyses were re-run in a number of ways. First we conducted separate correlations using data from each day of the study, controlling for preload energy intake in the case of Days 2 and 3. To increase power, we also combined cases by condition, such that separate correlations were conducted first for all children participating in the low energy preload condition on Day 2 or 3, then for all children participating in the high energy preload condition on Day 2 or 3. Re-conducting the analyses in each of these ways made no difference to the pattern of results.

In order that we might examine differences in intake between children who were more or less neophobic we divided participants at the median CFNS score (1.50) and compared means between low and high neophobia groups using independent t tests. The pattern of results was the same as for the correlational analyses, with the high neophobia group consuming less fruit and vegetables, protein and total calories than the low neophobia group (see Table 4).

**Table 4:** Mean (s.d.) intake of foods from 4 groups and total kilocalories consumed, by neophobia status

	**High neophobia (n = 52)**	**Low neophobia (n = 57)**
**Fruit and vegetables (g)**	30.81 (30.21)*	47.34 (38.96)*
**Protein (g)**	29.59 (23.54)**	47.66 (27.67)**
**Starch (g)**	29.87 (18.06)	24.65 (18.55)
**Snacks (g)**	48.32 (21.12)	49.72 (20.57)
**Total energy (kcal)**	405.33 (149.98)*	477.02 (159.34)*

Significance of difference: * p < 0.05, **p < 0.005

## Discussion

These results support the findings of previous research indicating that neophobia impacts differentially on consumption of different foods [7-9]. Specifically, children who scored highly on the Child Food Neophobia Scale ate less of foods in fruit and vegetable, and protein categories in a test meal as well as consuming fewer calories in total. This suggests that neophobic children may have less healthy diets overall than their less neophobic peers. These findings extend previous research by using direct observation of children's intake rather than relying on parent report.

The results are consistent with an evolutionary explanation for the trait of neophobia as a protective mechanism against the possibility of accidental poisoning [12] since plant and animal foods pose the most significant risk to children. However, although rejection of *unfamiliar *foods of these types is easily explicable in these terms, avoidance of *familiar *foods is less so, the latter being more characteristic of 'pickiness' than of neophobia. In all likelihood, children in the present study had previously encountered the *categories *of foods presented to them since all are commonly eaten by British children, although it is possible that they were unfamiliar with the particular *variety *of some or all of the foods offered.

Our results offer up the possibility that, notwithstanding the findings of Galloway and colleagues [9], neophobia and pickiness may be closely linked constructs and that the tendency to reject novel foods goes hand in hand with the tendency to reject less palatable, familiar foods. In the development of the Children's Eating Behaviour Questionnaire (CEBQ; [13]), Wardle and colleagues found that "food fussiness", a single factor encompassing both picky and neophobic behaviours emerged from Principal Components Analysis, supporting this proposal. It is also worth noting that despite achieving a Cronbachs' alpha of 0.92 in this sample, the six-item CFNS includes two items which would appear to measure picky, not neophobic behaviours: " My child is very particular about the foods he/she will eat" and "My child will eat almost anything" (reverse scored). Since research to date has failed to make the unfamiliar/familiar distinction when examining the relationship between neophobia and food intake, we cannot be sure that reported associations are not the result of confounding between neophobia and pickiness. Future research should examine the independent impact of neophobia and pickiness, if any, on willingness to try both familiar and unfamiliar foods in all major food groups, a task that would be facilitated by a more comprehensive measure of selective eating behaviours.

The finding of an inverse relationship between overall energy intake and neophobia requires discussion. It would appear that although neophobic children may have less healthy diets than their more neophilic peers in terms of the quantity of fruit and vegetables consumed, they do not compensate for their lower intake by eating more of foods in other categories; in this study, bread and snacks. These findings suggest that neophobia may be associated with lower risk of obesity, although we were unable to examine this possibility in the present study. The relationship between neophobia and adiposity in childhood warrants further investigation.

We acknowledge a number of limitations to the present study. Although the setting for the study was "natural" in the sense that the meals took place in schools at the usual time, children ate in their classrooms rather than in school dining rooms for the study period and the foods provided were not those usually served as part of school meals, although they were similar to typical packed lunches brought from home [14]. In addition, because this was part of a larger study of children's eating, children were aware of being observed by the researchers. Thus 'test' meals differed somewhat in content, procedure and atmosphere from normal school meals. Perhaps most importantly, lunches were preceded by low or high energy fruit flavoured soft drinks as part of a caloric compensation experiment. Nevertheless, there is no strong reason to hypothesize that relative consumption of different food types would be affected by this manipulation. Birch and colleagues [15] found no evidence for macronutrient-specific compensation in preschool aged children and the orange flavour drink used in the present study did not resemble any of the foods provided in the lunch, making any sensory-specific satiety effects unlikely. Finally, it would be helpful in future studies to include a measure of children's familiarity with and liking for the foods offered. This would permit us to evaluate whether differences in food intake observed are a product of simple preference rather than of neophobia.

## Conclusion

Whether in isolation or as part of a wider pattern of picky eating, our results suggest that neophobia is associated with less healthy food choices in children. Children with higher levels of neophobia as reported by their parents ate less fruit, vegetables and protein foods than their less neophobic peers. This study contributes to the existing literature in a number of ways. Firstly, in examining the relationship between parental rating of neophobia and actual mealtime food consumption, we have eliminated the problems of response bias and social desirability inherent in parental reports of children's food intake. Secondly, our findings highlight a lack of clarity that persists concerning classification of children's problematic eating behaviours and the need for better instruments with which to measure them. Finally, an important implication of our results is that attempts to increase intake of fruit, vegetables and protein could usefully incorporate strategies known to reduce the neophobic response, particularly modelling and taste exposure [16-19].

## Competing interests

The author(s) declare that they have no competing interests.

## Authors' contributions

LC developed the rationale for the study. LC and SC undertook data collection and statistical analysis. LC wrote the original draft and all three authors contributed to finalisation of the manuscript.
